# Nodal evaluation as the arbiter of oncologic equivalence: reassessing segmentectomy after CALGB 140503 and JCOG0802

**DOI:** 10.3389/fonc.2026.1986814

**Published:** 2026-10-09

**Authors:** Akbar Bazarbaev, Vasiliki Androutsopoulou, Prokopis-Andreas Zotos, Namariq Abbaker, Marco Scarci, Dimitrios E. Magouliotis

**Affiliations:** 1 Department of Surgery, Duke University Medical Center, Durham, NC, United States; 2 Department of Cardiothoracic Surgery, University of Thessaly, Larissa, Greece; 3 Department of Cardiothoracic Surgery, Hammersmith Hospital, Imperial College Healthcare Imperial College Healthcare National Health Service (NHS) Trust, London, United Kingdom; 4 Department of Cardiac Surgery Research, Lankenau Institute for Medical Research, Main Line Health, Wynnewood, PA, United States

**Keywords:** lobectomy, lymph node staging, non-small cell lung cancer, occult nodal metastasis, robotic-assisted thoracic surgery, segmentectomy, surgical quality

## Abstract

Two randomized trials, CALGB 140503 and JCOG0802, established segmentectomy as an oncologically acceptable alternative to lobectomy for small, peripheral, clinically node-negative non-small cell lung cancer. Both reached that conclusion under a protocol-enforced condition that is rarely emphasized when their results are applied in practice: nodal status had to be verified intraoperatively against a defined anatomic standard. CALGB 140503 required frozen-section confirmation of N0 status at two mediastinal stations and one major hilar node before randomization, whereas JCOG0802 mandated systematic or selective dissection of N1 and N2 stations in both arms and explicitly disallowed sampling as a substitute. This review examines whether that nodal rigor is reproduced, or even reported, once segmentectomy leaves the trial setting. National data indicate that it is not. Segmentectomy accounts for 4.4% of resections for clinical stage I to IIA disease, and only 12.6% of segmentectomy cases met all three Commission on Cancer quality measures, with adherence concentrated in academic and higher-volume programs. Retrospective series consistently report lower nodal upstaging after segmentectomy than after lobectomy, but the difference is driven by N1 rather than N2 stations and tracks the smaller nodal footprint of the operation, so a lower upstaging rate cannot by itself distinguish less disease from less looking. Anatomic mapping data indicate that exhaustive intersegmental dissection is unnecessary, whereas reliable hilar and mediastinal sampling is not. Across the wider literature, adequate nodal evaluation is defined inconsistently and reported at a granularity too coarse for readers to verify compliance, a two-axis problem that we characterize here for the segmentectomy literature specifically. Robotic assistance increases the number of nodal stations harvested relative to video-assisted surgery but has not been shown to improve risk-adjusted detection of occult disease. We propose a seven-point minimum standard, anchored on station-based rather than count-based adequacy, for segmentectomy performed outside a clinical trial.

## 1 Introduction

Lung cancer remains the leading cause of cancer-related mortality worldwide, with an estimated 2.48 million new cases and 1.8 million deaths recorded globally in 2022, accounting for 18.7% of all cancer deaths (1). Non-small cell lung cancer (NSCLC) accounts for approximately 85% of all lung cancer cases, with small cell lung cancer and rarer histologies making up the remainder (2), and among NSCLC subtypes adenocarcinoma has overtaken squamous cell carcinoma as the predominant histology worldwide (3). This histologic shift is not incidental to the question this review addresses: adenocarcinomas are more often peripheral and slower growing than squamous tumors, and it is precisely this subset of small, peripheral lesions that has driven renewed interest in resections less extensive than lobectomy. Management of NSCLC is heterogeneous and stage-dependent. Despite advances in detection, a substantial proportion of patients are still diagnosed at a locally advanced or metastatic stage, when resection alone is no longer curative (4, 5). For this group, stage III disease is generally managed with a multimodality approach combining chemotherapy and radiation, increasingly followed by consolidation immunotherapy, an approach that improved both progression-free and overall survival in the pivotal PACIFIC trial (6). Unresectable, oncogene-driven advanced disease is increasingly treated with targeted therapies and immune checkpoint inhibitors that have transformed outcomes over the past decade (2). In contrast, the minority of patients diagnosed early, with small, peripheral, node-negative tumors, are managed primarily with surgical resection, which remains the only potentially curative option in this population (2). It is specifically within this narrower early-stage surgical population that the choice of resection extent, lobectomy versus a parenchyma-sparing sublobar operation, and the rigor of the nodal evaluation underlying that choice, has been debated for three decades. That debate has never been simply a question of how much lung parenchyma to remove. At its core it is a question of how confidently a surgeon can rule out nodal disease before deciding that less tissue is enough.

Lobectomy became the default operation for early-stage NSCLC following the Lung Cancer Study Group randomized trial, which reported a 75% increase in overall recurrence among patients allocated to limited resection, attributable to a tripling of the local recurrence rate, together with a 30% increase in the overall death rate and a 50% increase in the rate of death with cancer (7). That result held surgical practice in place largely unchallenged for a generation, despite predating modern CT-based nodal staging, video-assisted surgical techniques, and any defined reporting standard for what adequate nodal evaluation should mean. Two developments arriving together eventually reopened the question. Low-dose CT screening, validated by the National Lung Screening Trial demonstration of a 20% relative reduction in lung-cancer-specific mortality (8), began detecting a different population of tumors than the one enrolled decades earlier: smaller, more peripheral, and often subsolid. The Japan Clinical Oncology Group JCOG0201 study then showed that the consolidation-to-tumor ratio (CTR) on thin-section CT could identify tumors unlikely to be pathologically invasive, with a specificity of 98.7% for radiologic noninvasiveness in adenocarcinoma no larger than 2.0 cm and a CTR no greater than 0.25 (9). For the first time, a preoperative radiological marker distinguished tumors that might be biologically quiet enough to treat with less than lobectomy. This radiological refinement mattered because it gave surgeons a defensible way to select which small tumors might tolerate a less extensive operation, rather than trusting that node-negative imaging alone was reassurance enough. Two randomized trials, CALGB 140503 and JCOG0802, then tested that possibility directly. Segmentectomy proved non-inferior to lobectomy on disease-free survival in CALGB 140503 (10), and, in JCOG0802, an overall survival advantage was additionally supported (11), although that trial was designed to test non-inferiority and its survival findings warrant the more cautious reading set out in Section 2.2, and both results have been widely read as settling the debate in favor of segmentectomy. Both trials, however, reached their conclusions under a specific, protocol-enforced condition, namely that nodal status be verified intraoperatively against a defined standard. It is that condition, rather than the extent of resection itself, that this review treats as the determining variable.

This review examines whether the oncologic equivalence demonstrated by CALGB 140503 and JCOG0802 depended on the rigor of their nodal evaluation protocols, and whether that same rigor is reflected in real-world segmentectomy practice and in the broader retrospective literature conducted outside these two trials. We further review the evidence on predictors of occult nodal upstaging, assess how inconsistently adequate nodal evaluation is defined and reported across the wider segmentectomy literature, which is the primary original contribution of this review, and evaluate whether robotic-assisted segmentectomy improves nodal dissection relative to video-assisted thoracoscopic surgery (VATS) or open approaches. We close with practical recommendations for what adequate nodal evaluation should mean when segmentectomy is offered outside a trial setting. Throughout, our emphasis is deliberately narrower than a general comparison of segmentectomy against lobectomy. We are not asking whether sublobar resection is oncologically sound in principle, since CALGB 140503 and JCOG0802 have already answered that question, but whether the nodal rigor that made their answer valid is being reproduced, or even reported, once segmentectomy leaves the trial setting.

## 2 Trial designs and nodal evaluation protocols in CALGB 140503 and JCOG0802

### 2.1 What a lymph node station is, and why the distinction matters

Intrathoracic lymph nodes relevant to lung cancer are not treated as an undifferentiated mass of tissue. They are mapped to specific, numbered anatomic sites under the International Association for the Study of Lung Cancer (IASLC) lymph node map, which groups fourteen numbered stations into supraclavicular, superior mediastinal, aortic, inferior mediastinal, and hilar, interlobar and peripheral zones (12). For staging purposes, those stations are further collapsed into three nodal compartments that determine the N category of a tumor and, in turn, its overall TNM stage. N1 nodes (stations 10 to 14) lie within the ipsilateral hilum, interlobar fissure, or lung parenchyma itself; N2 nodes (stations 1 to 9) lie in the ipsilateral mediastinum, including the subcarinal station 7; and N3 nodes lie in the contralateral mediastinum or hilum, or in the supraclavicular fossa, and confer unresectable disease by definition (13). This station-based architecture is what makes adequate nodal evaluation a meaningfully answerable question rather than a vague one. Because each station has a fixed anatomic identity, a protocol or a study can specify precisely which stations must be sampled or dissected, and a reader can verify whether it was. That precision is the reason this review is organized around station-level detail rather than aggregate node counts, and it is the same architecture that **Figure 1** depicts and that CALGB 140503, JCOG0802, and the guideline standards discussed throughout this review each reference when they define their own nodal evaluation requirements.

**Figure 1:**
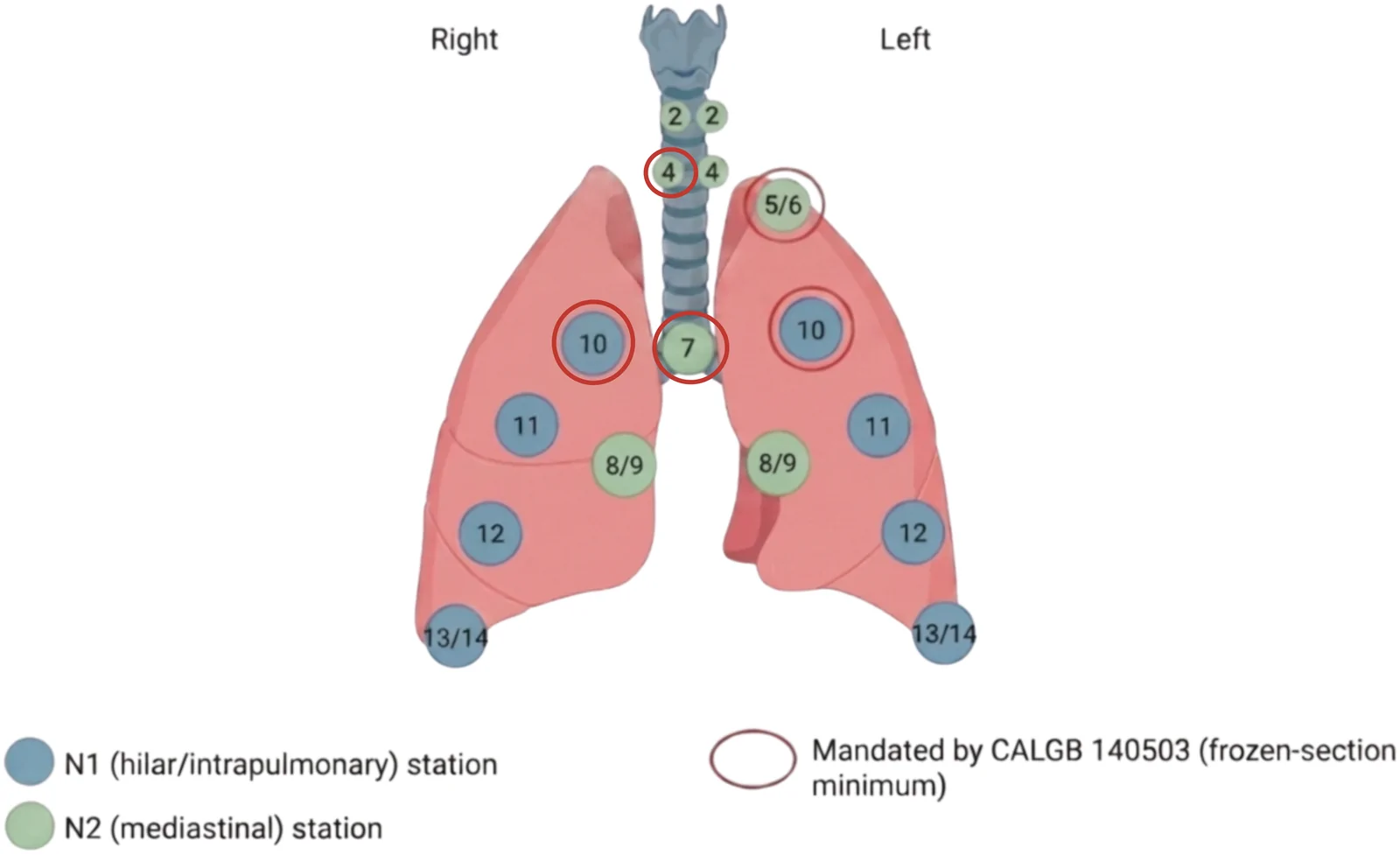
Lymph node stations relevant to segmentectomy and the varying station-level requirements across nodal evaluation standards. Ringed stations denote the frozen-section pre-randomization minimum of CALGB 140503 (stations 4, 7 and 10 on the right; stations 5 or 6, 7 and 10 on the left) (10). JCOG0802 mandates dissection of the full N1 and N2 field shown (11). Current Commission on Cancer Operative Standard 5.8 requires at least one N1 and three N2 stations but does not specify which (15). Created in BioRender. Magouliotis, D. (2026) https://BioRender.com/ogurie2.

### 2.2 Two trials, two nodal standards

Two independent randomized controlled trials established segmentectomy as an oncologically acceptable alternative to lobectomy for selected patients with small, peripheral, clinically node-negative NSCLC: the Alliance/CALGB 140503 trial (10) and the Japanese JCOG0802/WJOG4607L trial (11). Although both reached broadly concordant conclusions, they differed materially in geography, eligibility, primary endpoint, and, most importantly for this review, in how and when nodal status was verified.

CALGB 140503 enrolled 697 patients, from 1080 registered, with peripheral clinical T1aN0 NSCLC no larger than 2 cm across 69 academic and community sites in the United States, Canada, and Australia (10). Nodal evaluation was performed prospectively, before randomization: intraoperative frozen section examination was required to confirm the absence of nodal metastases at a minimum of two mediastinal stations and one major hilar node, a strategy the trial termed simple sampling, specifically stations 4, 7, and 10 for right-sided tumors and stations 5 or 6, 7, and 10 for left-sided tumors (10). Only after this frozen-section confirmation of N0 status were patients randomized to lobar or sublobar resection. Additional nodal evaluation, in the form of systematic sampling of three or more mediastinal stations or complete mediastinal lymphadenectomy, was permitted at the discretion of the operating surgeon but was not mandatory beyond this minimum (10). A secondary analysis of the trial found that once this station-based minimum was met, the extent of additional dissection was not associated with disease-free survival: five-year disease-free survival was 62.3% after complete lymph node dissection, 65.7% after systematic sampling, and 61.2% after simple sampling (14). That finding is consistent with station coverage, rather than absolute nodal count or dissection extent, having governed oncologic safety in this trial, but three limits qualify how far it can be carried. The comparison was not randomized: the extent of dissection beyond the mandated minimum was left to the operating surgeon, so the three groups were defined by intraoperative judgment rather than by allocation, and residual confounding by indication cannot be excluded. Nodal upstaging was not a prespecified endpoint of the trial, and the analysis was not powered to detect a difference in it. The trial investigators themselves restrict the finding to a cohort rendered node-negative by meticulous radiographic and intraoperative staging (14). Applied to a population selected less strictly, the same result could be read as licensing a lighter dissection than the trial ever tested, with a corresponding risk of incompletely staged resections and of understaging that removes the opportunity for adjuvant therapy. We therefore read it as evidence that the station-based floor was sufficient in this closely staged cohort, not as evidence that dissection beyond that floor is unnecessary in general.

Two further features of the CALGB 140503 population limit direct transfer of its nodal findings to segmentectomy specifically. First, the sublobar arm was not a segmentectomy arm. Of 340 patients assigned to sublobar resection, 201 (59.1%) underwent wedge resection and 129 (37.9%) an anatomical segmentectomy (10), so the nodal experience of the trial is dominated by an operation that does not follow segmental planes at all and that removes no intersegmental nodal tissue by design. Second, the radiological composition of the cohort is incompletely characterized. Pure ground-glass lesions were excluded, but the proportion of part-solid tumors among those enrolled was not reported, whereas in JCOG0802 more than 90% of patients had adenocarcinoma and 45% of those had a ground-glass component. The CALGB investigators attribute part of the survival difference between the two trials to precisely this imbalance, noting that distant metastases developed in 16.0% of their own patients against roughly 5% in JCOG0802 (10). Comparisons between the two trials, and extrapolation from either to the case mix of a given institution, should be read with that difference in mind.

JCOG0802 took a categorically different approach. The trial randomized 1106 patients with peripheral tumors no larger than 2 cm and a CTR greater than 0.5 across Japanese centers to segmentectomy (n=552) or lobectomy (n=554) (11). Rather than a minimum pre-randomization sampling gate, the protocol mandated formal systematic or selective lymph node dissection, encompassing hilar, interlobar, and intrapulmonary (N1) stations as well as mediastinal (N2) stations, as part of the definitive operation itself, applied identically in both arms; nodal sampling alone was explicitly disallowed as a substitute for dissection (11). On its primary endpoint, five-year overall survival was 94.3% after segmentectomy and 91.1% after lobectomy, and a prespecified superiority test was met (hazard ratio 0.663, 95% CI 0.474 to 0.927; *p* = 0.0082 for superiority) (11). That result should nonetheless be read within the non-inferiority framework the trial was built on, and alongside its other endpoints. Relapse-free survival was essentially identical between the arms (88.0% versus 87.9%; hazard ratio 0.998, 95% CI 0.753 to 1.323), and local relapse was roughly twice as frequent after segmentectomy (10.5% versus 5.4%; *p* = 0.0018) (11). The overall survival advantage is therefore not explained by better locoregional cancer control, and deaths from other diseases accounted for a substantial share of events in both arms. For the purposes of this review, JCOG0802 is best read as supporting the oncologic acceptability of segmentectomy performed under mandatory nodal dissection, rather than as demonstrating that segmentectomy confers a survival benefit. This design difference matters. JCOG0802 never permits a segmentectomy without dissection, whereas the minimum requirement of CALGB 140503 could in principle be satisfied without ever escalating beyond simple sampling. In practice, the two trials therefore answer subtly different questions: JCOG0802 demonstrates that segmentectomy is safe when dissection is mandatory and uniform, while CALGB 140503 demonstrates that it can be safe with a considerably lighter, sampling-based floor. The two results are not automatically interchangeable evidence for a surgeon deciding how much nodal work a given segmentectomy actually requires. These design differences are summarized in **Table 1**.

**Table 1:** Trial design and nodal evaluation protocols in CALGB 140503 and JCOG0802.

Parameter	CALGB 140503 (10)	JCOG0802 (11)
Sites and sample size	69 sites in the United States, Canada and Australia; 1080 registered, 697 randomized (340 sublobar, 357 lobar)	Japan; 1106 randomized (552 segmentectomy, 554 lobectomy)
Eligibility	Peripheral solid tumor ≤2 cm, cT1aN0	Peripheral tumor ≤2 cm, CTR >0.5
Timing of nodal evaluation	Pre-randomization, intraoperative, before allocation	Intraoperative, during definitive resection, both arms
Minimum nodal requirement	Frozen-section N0 confirmation at ≥2 mediastinal stations (4, 7, 10 right; 5 or 6, 7, 10 left) plus 1 major hilar node (simple sampling)	Mandatory systematic or selective dissection of N1 and N2 stations; sampling alone not permitted
Escalation beyond minimum	Surgeon discretion (systematic sampling of ≥3 stations, or complete dissection); extent not associated with disease-free survival (14)	Not applicable; dissection uniformly mandated
Primary endpoint	Disease-free survival	Overall survival
Key result	Non-inferior disease-free survival for sublobar resection	Non-inferior overall survival; prespecified superiority test also met (HR 0.663; *p* = 0.0082), with equivalent relapse-free survival and higher local relapse (10.5% vs 5.4%)

### 2.3 How trial standards compare with guidelines and with real-world practice

Despite their differences, both protocols share a feature directly relevant to real-world translation: neither trial validated segmentectomy against a nodal evaluation standard weaker than contemporary guideline recommendations. Current consensus among the National Comprehensive Cancer Network (NCCN), the European Society of Thoracic Surgeons (ESTS), the IASLC, and the American College of Surgeons Commission on Cancer (CoC), codified in CoC Operative Standard 5.8, defines adequate intraoperative nodal evaluation as sampling of at least one N1 (hilar or intrapulmonary) station and three distinct N2 (mediastinal) stations (15, 16). The minimum requirement of CALGB 140503 sits close to, but is structurally different from, this threshold, requiring two mediastinal stations and one hilar node rather than three and one, while the mandatory dissection requirement of JCOG0802 exceeds it in practice.

Real-world practice does not consistently meet either bar. A National Cancer Database analysis of 249,391 patients undergoing resection for clinical stage I to IIA NSCLC between 2004 and 2018 found that segmentectomy accounted for only 4.4% of resections nationally (n=11,006), and that the proportion of resections performed as segmentectomy nearly doubled over the study period, from 3.3% in 2004 to 6.1% in 2018 (17). Adoption rose, but quality did not keep pace: only 12.6% of segmentectomy patients met all three CoC quality measures, which at the time comprised retrieval of at least 10 lymph nodes, negative margins, and tumor size no greater than 2 cm, and adherence was significantly more likely at academic than at non-academic programs (odds ratio 1.56, 95% CI 1.14 to 2.15). Adherence to all three measures was associated with improved survival (hazard ratio 0.67, 95% CI 0.56 to 0.79) (17). A separate national analysis of 253,182 patients across 1324 hospitals corroborates this institutional pattern: lymph node sampling was performed in 91% of patients nationally, yet only 76% of hospitals reached an 85% adherence threshold, and higher-volume academic centers showed significantly higher adherence to surgical quality measures than lower-volume or community centers even as their performance on care coordination measures was sometimes worse (18). This institution-dependent adherence gap is the empirical crux of the argument of this review. The nodal rigor that made segmentectomy provably safe in CALGB 140503 and JCOG0802 is itself unevenly distributed among those now adopting segmentectomy on the strength of those trials. The gap is not a matter of a handful of outlier institutions falling short. Even at its most improved, national adherence leaves the large majority of segmentectomy cases short of a fully adequate standard, which means the safety margin of the trials is being extended, in practice, to a population that was never actually studied at that margin.

**Figure 1** grounds these protocol differences anatomically. Mapped onto the IASLC lymph node station scheme, the frozen-section minimum of CALGB 140503 corresponds to a specific, named subset of stations, namely 4, 7 and 10 on the right and 5 or 6, 7 and 10 on the left (10), while the mandate of JCOG0802 covers the full N1 and N2 field shown (11), and the current CoC Operative Standard 5.8 requires a minimum count of at least one N1 and three N2 stations without naming specific stations at all (15). The same anatomic map that defines where disease can hide is not applied identically by any two of these standards.

## 3 Occult nodal upstaging after segmentectomy and lobectomy: rates and predictors

Retrospective series consistently report a lower rate of pathologic nodal upstaging after segmentectomy than after lobectomy, but the magnitude of that gap, and how much of it reflects a true biological difference rather than dissection extent, varies by study. In the largest recent single-institution comparison, Suzuki and colleagues reviewed 957 lobectomies and 402 segmentectomies performed for clinical T1N0M0 NSCLC no larger than 3 cm between 2012 and 2022 (19). Occult N1 disease was detected in 13.3% of lobectomy patients versus 3.7% of segmentectomy patients (*p* < 0.001), whereas the difference in occult N2 disease was not statistically significant (5.5% versus 3.2%, *p* = 0.074). The median number of lymph nodes sampled was correspondingly higher after lobectomy (18 versus 12, *p* < 0.001), and the gap was widest for intrapulmonary nodes retrieved by the pathologist from the resected specimen (median 4 versus 0, *p* < 0.001). The location of that gap is informative: the upstaging difference was concentrated precisely in the nodal compartment that segmentectomy removes less of, and largely absent in the mediastinal compartment that both operations are expected to address independently of parenchymal extent.

This pattern, lower upstaging paired with lower nodal yield, recurs throughout the literature and is the central methodological problem for this section. A lower observed upstaging rate after segmentectomy cannot, by itself, distinguish less disease to find from less looking. Disentangling these two possibilities matters clinically and not only methodologically. If segmentectomy patients genuinely carry less nodal disease because they are selected for smaller and more indolent tumors, a lower upstaging rate is reassuring; if the lower rate simply reflects a smaller dissection footprint, some fraction of segmentectomy patients are being understaged and, by extension, undertreated. Two additional analyses reproduce the same pattern in independent data. In a National Cancer Database cohort of 22,945 patients with clinical T1cN0M0 disease, pathologic upstaging to pN1 or higher occurred in 14.5% of lobectomy patients versus 6.6% of segmentectomy patients, yet propensity-matched overall survival was equivalent between the two operations (hazard ratio 1.00, 95% CI 0.86 to 1.16), and remained equivalent among the subset found to have occult pN1 disease (hazard ratio 1.04, 95% CI 0.65 to 1.66) (20). Similarly, in a propensity-weighted single-institution analysis of 185 patients with occult pN1 disease, of whom 30 underwent segmentectomy and 155 lobectomy, Abdallat and colleagues found no difference in five-year overall survival or locoregional recurrence-free survival between the two operations, and procedure type was not a predictor of outcome on multivariable analysis (21). Taken together, these data suggest that upstaging rate and oncologic benefit are related but not identical questions, and that the clinical cost of a missed N1 node in a well-selected patient may be smaller than the staging arithmetic alone implies.

Station-specific data help resolve the ambiguity. Matsumura and colleagues examined 307 patients who underwent lobectomy with systematic nodal dissection for tumors that would have been anatomically resectable by segmentectomy, and mapped where nodal metastases actually occurred relative to segmental boundaries (22). Of the 307 patients, 34 (11%) proved node-positive. Nine of these had metastasis in the lobar-segmental nodes that a segmentectomy cannot reliably reach, which Matsumura and colleagues designate isolated lobar-segmental nodes and locate at station 11 in five patients and station 13 in four. In every one of those nine, disease was also present at station 12 or in the mediastinum, and no patient in the series had a solitary metastasis confined to an isolated lobar-segmental node. All 34 node-positive patients had solid-dominant tumors on thin-section CT, with a tumor disappearance rate below 0.25, which ties the anatomic finding to the same radiological selection discussed above (22). This is the strongest anatomic argument that adequate nodal evaluation at segmentectomy does not require exhaustive intersegmental dissection, but does require reliable hilar and mediastinal sampling, which are the same stations that both CALGB 140503 and JCOG0802 mandated. The anatomic data and the trial protocols converge on the same answer from two independent directions, one describing where disease actually travels and the other describing where trial designers chose to look, which strengthens confidence that the overlap is not coincidental.

### 3.1 Predictors of occult nodal metastasis

Across the predictive modeling literature, four variables recur as independent predictors of occult nodal disease.

Tumor size. Tumor size is consistently identified as an independent predictor of nodal metastasis, both as a continuous variable and above threshold cutoffs, across nomogram and predictive model studies (23, 24).

Consolidation-to-tumor ratio. Zhao and colleagues, analyzing 215 training and 161 validation patients with part-solid stage IA adenocarcinoma, found microvascular invasion, micropapillary or solid histologic subtype, and CTR greater than 0.61 to be independently associated with nodal metastasis (23). Two of those three variables are pathologic rather than preoperative, and the model was designed to be applied with frozen-section input during the operation rather than from imaging alone, which limits its use for preoperative selection. Li and colleagues, in a 435-patient cohort restricted to tumors no larger than 2 cm with CTR greater than 0.5, and therefore already at higher radiologic risk, identified tumor size, CTR, pleural indentation, and carcinoembryonic antigen (CEA) as four independent predictors in a model externally validated at a second institution (area under the curve 0.890) (24). JCOG0802 itself used a CTR greater than 0.5 as an eligibility criterion (11), so the predictive literature and the selection criteria of the trial point at the same radiologic variable from different directions.

Pleural indentation, CEA, and composite nomograms. Ai and colleagues developed and case-matched a clinical nomogram combining CEA, CTR, mean CT attenuation, and tumor margin to predict occult nodal metastasis in NSCLC no larger than 2 cm with a solid component, achieving a cross-validated area under the curve of 0.86 (25). The performance of these composite models reinforces that no single variable is sufficient and that multivariable risk stratification outperforms tumor size or CTR alone.

Tumor location. Location-specific data are more substantial than a passing qualitative mention. Defining centrality as location in the inner third of the hemithorax, one series found occult N2 disease in 21.1% of central tumors versus 6.2% of peripheral tumors, though a looser inner-two-thirds definition narrowed this gap considerably, to 8.8% versus 7.4%, underscoring how sensitive this predictor is to definition (26). Lobar location also matters: right lower lobe and right upper lobe tumors carried the highest incidence of N2 disease, 30% and 27% respectively, in one distribution analysis, and lobe-specific dissection protocols now propose that upper lobe tumors may not require lower mediastinal nodal dissection, and lower lobe tumors may not require upper mediastinal dissection (27). A recent methodological review nonetheless cautions that location-based predictors are less standardized across studies than size-based or CTR-based ones, which limits direct comparison (28).

Taken together, these four predictors describe a risk profile rather than a single decisive test. No one variable reliably rules occult nodal disease in or out, which is itself an argument for weighing several criteria together rather than relying on preoperative imaging alone to justify skipping intraoperative nodal assessment. Representative studies are summarized in **Table 2**, **Figure 2**.

**Table 2:** Occult nodal upstaging: representative studies, predictors, and station-level findings.

Study	Design and sample	Upstaging rate	Predictors identified	Station-level finding
Suzuki et al, 2025 (19)	Retrospective, single center; 402 segmentectomy, 957 lobectomy; cT1N0M0 ≤3 cm	Occult N1 3.7% vs 13.3% (*p* < 0.001); occult N2 3.2% vs 5.5% (*p* = 0.074)	Not assessed	Median nodes 12 vs 18 (*p* < 0.001); intrapulmonary specimen nodes 0 vs 4 (*p* < 0.001)
Jacobs et al, 2025 (20)	NCDB; 22,945 patients, cT1cN0M0 (1070 segmentectomy, 21,875 lobectomy)	pN1 + 6.6% vs 14.5%	Not assessed	Matched overall survival equivalent (HR 1.00), including within pN1+ subset (HR 1.04)
Abdallat et al, 2026 (21)	Single center, propensity weighted; 185 patients with occult pN1 (30 segmentectomy, 155 lobectomy)	Cohort restricted to upstaged patients	Not assessed	No difference in 5-year overall or locoregional recurrence-free survival
Matsumura et al, 2012 (22)	Retrospective; 307 lobectomy with systematic dissection (segmentectomy-candidate anatomy)	Anatomic mapping study	Not assessed	9 of 34 node-positive cases had isolated lobar-segmental node involvement (station 11 n=5, station 13 n=4); all 9 also had station 12 or mediastinal metastasis, none solitary
Zhao et al, 2022 (23)	Retrospective; 215 training, 161 validation; part-solid stage IA adenocarcinoma	Not reported	Microvascular invasion, micropapillary or solid subtype, CTR >0.61	Not assessed
Li et al, 2025 (24)	Retrospective; 435 patients, ≤2 cm and CTR >0.5, externally validated	Not reported	Tumor size, CTR, pleural indentation, CEA (AUC 0.890)	Not assessed
Ai et al, 2024 (25)	Retrospective, case matched; NSCLC ≤2 cm with solid component	Not reported	CEA, CTR, mean CT attenuation, tumor margin (AUC 0.86)	Not assessed
Roy et al, 2020 (26)	Retrospective; cT1N0M0 cohort	Occult N2 21.1% central vs 6.2% peripheral (inner-third definition)	Tumor centrality (definition sensitive)	Not assessed
Cerfolio and Bryant, 2006 (27)	Retrospective, lobe-stratified cohort	N2 incidence highest in right lower lobe (30%) and right upper lobe (27%)	Lobar location	Supports lobe-specific dissection extent

**Figure 2:**
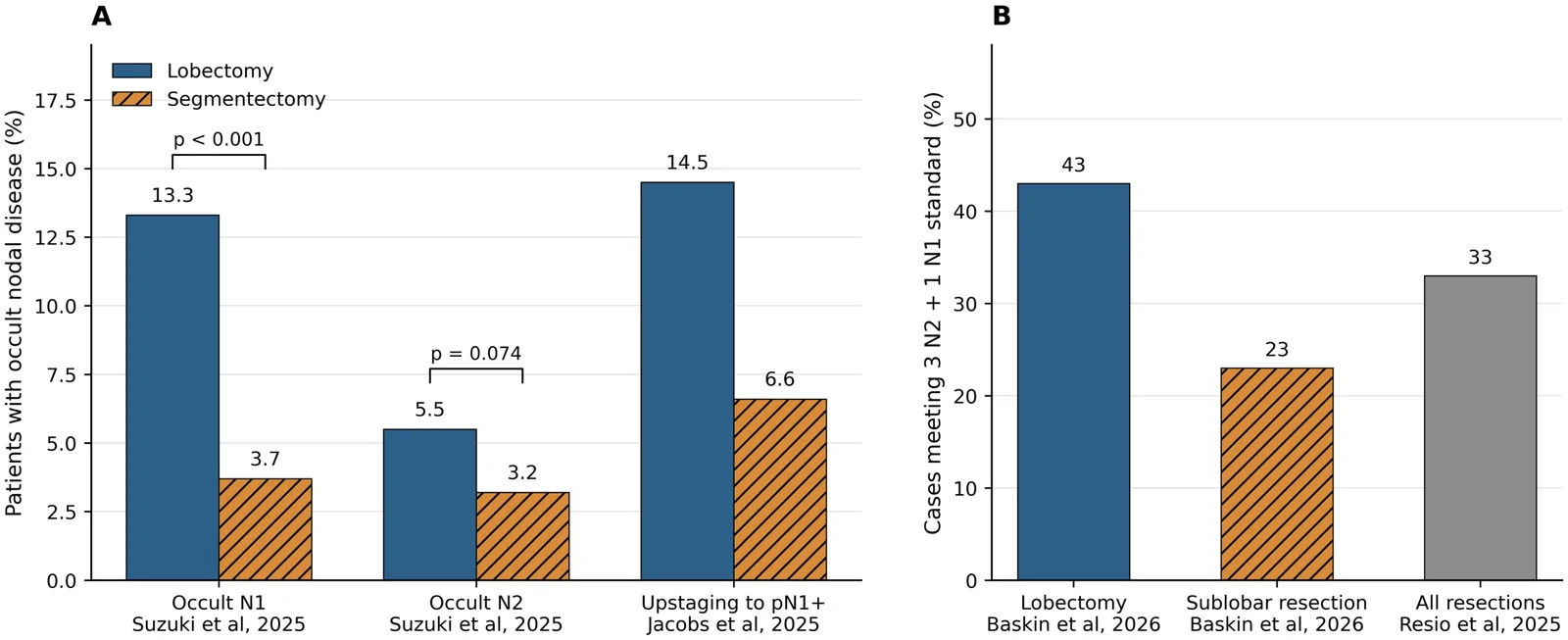
Occult nodal upstaging and compliance with the station-based nodal standard, by extent of resection. **(A)** Occult N1 disease was more frequent after lobectomy than segmentectomy (13.3% versus 3.7%, *p* < 0.001), whereas the occult N2 difference was not significant (5.5% versus 3.2%, *p* = 0.074), in 957 lobectomies and 402 segmentectomies for cT1N0M0 tumors ≤3 cm. (19) Upstaging to pN1 or higher was 14.5% versus 6.6% in an independent cohort of 22,945 patients with cT1cN0M0 disease; no p value was reported. (20) **(B)** Cases meeting Commission on Cancer Operative Standard 5.8 (≥1 N1 and ≥3 N2 stations): 43% of lobectomies and 23% of sublobar resections, (35) and 33% of 9,289 consecutive resections in a separate series. (15) Panel B combines two independent cohorts and is not a direct statistical comparison. Created in BioRender. Magouliotis, D. (2026) https://BioRender.com/ea68qy0.

## 4 Heterogeneity in defining adequate nodal evaluation outside the two trials

CALGB 140503 and JCOG0802 each specified, unambiguously, what nodal evaluation had to occur before their conclusions could apply (10, 11). Outside these two protocols, the segmentectomy literature has no such consensus, and the inconsistency operates on two separate axes that are frequently conflated: what counts as adequate, which is the definitional threshold, and how that adequacy is reported, which is the granularity of what studies actually publish. Neither axis has been systematically mapped for the segmentectomy-specific literature, which is the gap this review addresses. The distinction is worth dwelling on because the two axes fail independently. A study can use a rigorous, station-based definition of adequacy and still report it so vaguely that a reader cannot verify compliance, just as a study can report granular station-level detail while applying a definition of adequacy that would not satisfy current guideline standards. Closing only one gap without the other leaves the literature no more auditable than before.

### 4.1 Definitional heterogeneity

Even among authoritative bodies, the operational definition of adequate nodal evaluation has shifted substantially over time and continues to vary by source. For decades, the CoC quality metric was an absolute count: retrieval of 10 or more lymph nodes from anywhere in the chest, with no requirement on which stations they came from (29). Contemporary guidance has moved away from this toward a station-based standard. CoC Operative Standard 5.8 now requires sampling of at least one N1 station and three distinct N2 stations, a definition converged upon by the NCCN (30), ESTS (31), and IASLC as well (15, 16). The trial-specific minimum of CALGB 140503, namely one hilar node plus two mediastinal stations confirmed by frozen section before randomization, is close to but not identical to the current CoC standard (10). JCOG0802 differs categorically again, mandating dissection rather than any sampling-based threshold (11). That five credible, contemporaneous sources, comprising the historical CoC metric, the current CoC standard, the NCCN and ESTS recommendations, and the two trials, do not converge on a single identical definition of adequacy is itself evidence that no single benchmark currently governs the field.

Terminological heterogeneity compounds this. The literature does not use sampling and dissection consistently. Systematic sampling, lobe-specific systematic dissection, and complete mediastinal lymphadenectomy are sometimes used interchangeably despite describing different amounts of tissue removed, and a technique labeled lobe-specific systematic dissection in one series may, on closer inspection, meet only the definition of systematic sampling (29, 32). This is not a semantic quibble. It means that when two retrospective segmentectomy series both report systematic nodal evaluation, they may not have performed comparable procedures, and pooled or narrative synthesis across such series risks averaging together operations that differ in oncologic rigor. This is a familiar hazard in surgical evidence synthesis more broadly, but it is particularly consequential here, because extending trial-level safety data to real-world practice depends on the assumption that nodal evaluation means roughly the same thing across studies, an assumption the terminological inconsistency documented here does not support.

### 4.2 Reporting heterogeneity

Reporting heterogeneity is the more consequential problem for evidence synthesis. Existing systematic reviews and meta-analyses of segmentectomy versus lobectomy, conducted for purposes other than auditing nodal adequacy, have already flagged this as a limitation without resolving it. The 2023 meta-analysis by Dai and colleagues of segmentectomy versus lobectomy for stage IA NSCLC notes that heterogeneity in the extent of mediastinal lymph node dissection across pooled studies contributes to variability in reported upstaging rates and survival outcomes (33). The meta-analysis by Bertolaccini and colleagues similarly reports harvested lymph node count as the primary nodal comparator between minimally invasive lobectomy and segmentectomy, without a station-based adequacy threshold applied to the pooled cohorts (34). In both cases the authors treat nodal evaluation heterogeneity as background noise to control for statistically rather than as a primary finding to characterize. That is a reasonable choice for a meta-analysis whose primary outcome is survival or recurrence, but it is precisely the choice this review declines to make, since our interest is in the adequacy of nodal evaluation itself rather than in adjusting around it.

The stakes of this inconsistency are not merely academic, although the evidence on whether any single threshold improves outcomes is itself mixed. In a prospective institutional series of 9289 pulmonary resections, only 33% were concordant with the three-N2-plus-one-N1 standard; concordance was associated with higher nodal upstaging (21% versus 13%) and more in-hospital complications, but not with reduced recurrence or improved survival in clinical stage I or II disease, with a survival benefit apparent only in clinical stage III (15). Baskin and colleagues, however, found that meeting the same threshold was associated with reduced one-year recurrence after sublobar resection specifically (adjusted hazard ratio 0.62, 95% CI 0.39 to 0.98) while showing no significant association with recurrence or five-year mortality after lobectomy, and that only 23% of sublobar resections met the threshold compared with 43% of lobectomies (35). Read together, these two studies suggest that a single nodal adequacy threshold does not carry uniform prognostic weight across resection types, and that the resection type for which it appears to matter most is precisely the one that meets it least often. Segmentectomy-specific adequacy standards may therefore need to be validated separately rather than inherited wholesale from lobectomy-era metrics. Facility-level compliance with even the current CoC standard varies with institutional volume and academic status (17, 18), which means that whatever definition of adequacy a given series claims to follow, actual practice may fall short of it in ways that are invisible without the station-level audit data that most publications do not report. **Table 3** juxtaposes the operational definitions found across the sources reviewed above.

**Table 3:** Operational definitions of adequate nodal evaluation across sources.

Source	Definition of adequate evaluation	Unit of measurement	N1 and N2 distinguished
Historical ACS/CoC standard (pre-2021) (29)	≥10 lymph nodes, any station	Absolute count	No
Current CoC Operative Standard 5.8 (15)	≥1 N1 station plus ≥3 N2 stations	Station based	Yes
NCCN version 7.2025 (30)	Station-based mediastinal evaluation; subcarinal node inclusion emphasized	Station based	Yes
ESTS 2023 technical consensus (31)	Systematic or lobe-specific hilar and mediastinal dissection; specific guidance on management of unexpected positive N1 or N2 disease	Station based, dissection oriented	Yes
CALGB 140503, trial-specific minimum (10)	≥1 hilar node plus ≥2 mediastinal stations, frozen-section confirmed before randomization	Station-based minimum	Yes
JCOG0802, trial-specific mandate (11)	Mandatory systematic or selective dissection of N1 and N2; sampling alone disallowed	Dissection, not sampling	Yes
Typical retrospective segmentectomy series (33, 34)	Frequently unspecified, or reported only as mean or median total node count	Absolute count most common; station-level detail often absent	Inconsistent, often not distinguished

### 4.3 Toward a systematic characterization

The next step, beyond the scope of this narrative synthesis but a natural extension of it, would be a structured audit of the broader segmentectomy literature, covering for example all comparative segmentectomy and lobectomy series published since JCOG0802 and CALGB 140503, coded against four criteria: whether a nodal evaluation method is specified at all; whether adequacy is reported as absolute node count, station count, or both; whether N1 and N2 findings are reported separately; and whether the study explicitly benchmarks its own nodal evaluation against a named external standard such as CoC Operative Standard 5.8, the NCCN guidelines, or the ESTS consensus. We propose this framework both as a diagnostic of the shortcomings of the current literature and as a template for how future segmentectomy series should report their nodal evaluation.

## 5 Robotic-assisted segmentectomy and nodal evaluation

A consistent, modest signal in the literature suggests that robotic assistance improves nodal yield relative to VATS. In a series of 717 resections, Hurley and colleagues found a statistically higher mean nodal harvest with robotic assistance than with VATS (6.1 plus or minus 1.5 versus 5.53 plus or minus 1.8 nodes, p=0.002), along with more N1 stations harvested (2.66 versus 2.36) (36). At the meta-analytic level, Ma and colleagues pooled 18 studies comprising 11,247 patients across lobectomy and segmentectomy cohorts and likewise found robotic-assisted surgery associated with more harvested lymph nodes and more nodal stations examined relative to VATS (37). A more recent single-institution propensity-matched comparison of robotic versus VATS segmentectomy by Agyabeng-Dadzie and colleagues refines this picture: after matching 102 robotic to 204 VATS cases, the robotic group had a significantly greater median number of nodal stations harvested, but no significant difference in the median number of lymph nodes, and no difference in three-year disease-free or overall survival (38). The proposed mechanism is plausible on technical grounds, in that articulated instrumentation and enhanced three-dimensional visualization are argued to ease dissection along the intersegmental plane and around the hilum, where anatomic planes are less forgiving than in a standard lobar dissection (39).

Whether this yield advantage translates into meaningfully better detection of occult N1 or N2 disease is a separate question, and the evidence here is thinner and less consistent. Alaparthi and colleagues found in a retrospective cohort that robotic segmentectomy was associated with both a higher number of nodes examined and higher rates of nodal upstaging compared with VATS on unadjusted analysis, but noted that some of this difference attenuated on adjusted analysis, leaving open how much of the raw upstaging difference reflects genuinely improved detection rather than case mix or surgeon and institutional selection effects (40). Pointing the other way, the same series that reported the highest robotic nodal yield found nodal upstaging from N0 or N1 to N2 in 6.4% of robotic cases versus 8.2% of VATS cases, that is, numerically more upstaging in the arm that harvested fewer nodes (36). No study identified in this review reports occult N1 or N2 detection as a prespecified, risk-adjusted primary outcome comparing robotic to VATS or open segmentectomy specifically; the claim of a detection advantage is inferred from yield differences rather than demonstrated directly.

The distinction matters because more lymph nodes or stations examined is a process measure, while occult disease detected is the outcome measure that actually bears on staging accuracy and adjuvant treatment decisions, and the two are not interchangeable. The narrative review by Fortich and colleagues argues that robotic instrumentation offers intraoperative advantages specifically for intersegmental and segment-specific dissection in complex segmentectomy (39), but this claim rests on technique description and case series experience rather than on a comparative, outcome-adjusted study designed to isolate detection rate as the endpoint. On the available evidence, robotic assistance confers a real if modest technical advantage in nodal yield during segmentectomy, particularly in the number of stations reached. It does not yet support a confident claim that this advantage meaningfully improves detection of occult nodal disease once case selection and surgeon experience are accounted for.

## 6 What adequate nodal evaluation should mean outside a trial setting

The preceding sections point toward a consistent conclusion. The oncologic equivalence of segmentectomy to lobectomy was established under specific, protocol-enforced nodal evaluation conditions (10, 11), conditions that current guideline bodies have largely converged on but that real-world practice meets unevenly (15, 17), and that surgical approach does not by itself guarantee (37). Translating this into practice requires moving from description to a proposed minimum standard. None of the seven recommendations below requires new equipment, a new trial, or a change in operative technique. Each asks only that information already available, whether a preoperative CTR value or an intraoperative station count, be documented and checked against an explicit threshold rather than left to individual judgment.

### 6.1 Preoperative risk stratification should be documented, not assumed

Tumor size, CTR, and, where feasible, tumor location relative to the hilum should be recorded, since all three are validated preoperative predictors of occult nodal disease (23, 24, 26, 27), and a CTR greater than 0.5 is the threshold JCOG0802 itself used for eligibility (11). Before segmentectomy is offered, these variables should be explicitly documented, and centers should have a defined, if institution-specific, threshold above which additional preoperative nodal staging with endobronchial ultrasound or mediastinoscopy is considered rather than proceeding directly to resection.

### 6.2 Intraoperative nodal evaluation should meet a station-based, not count-based, minimum

Consistent with the current CoC Operative Standard 5.8, the NCCN, and the ESTS (15, 30, 31), we propose that segmentectomy offered outside a trial should require sampling or dissection of at least one N1 station and three distinct N2 stations, explicitly including station 7. This sits at or above the minimum used in CALGB 140503 (10) and is consistent with the more stringent mandatory dissection requirement of JCOG0802 (11). Anatomic mapping data suggest that exhaustive dissection of the isolated lobar-segmental nodes specifically is not required to meet this standard, since in the available evidence no patient had a solitary metastasis at such a node without concurrent station 12 or mediastinal involvement (22).

### 6.3 The choice between sampling and dissection is secondary to station coverage

A secondary analysis of CALGB 140503 found no association between dissection extent and disease-free survival once the station-based minimum of the trial was met (14). As set out in Section 2.2, that analysis was not randomized for dissection extent and describes a cohort staged more rigorously than routine practice, so we intend this recommendation as permission to match dissection to the anatomic difficulty of a case once the floor is met, not as a statement that the floor is also a ceiling. This supports a coverage-first standard, in which what matters is that the specified stations are examined rather than whether they are addressed by sampling or by complete lymphadenectomy, though the design of JCOG0802 and the ESTS consensus on managing unexpected positive nodes suggest that dissection remains the more conservative default where feasible (11, 31). In practice this leaves room for surgeons to match the extent of nodal work to the anatomic difficulty of a given case, provided the specified stations are still examined, rather than treating sampling and dissection as a binary choice fixed at the start of the operation.

### 6.4 A frozen-section confirmation gate is advisable before finalizing segmentectomy

The design of CALGB 140503, confirming N0 status intraoperatively before committing to the sublobar extent of resection, offers a practical template. If nodal stations sampled intraoperatively return positive, conversion to lobectomy or completion of a more extensive dissection should be the default, rather than proceeding with segmentectomy on the basis of preoperative imaging alone (10). **Figure 3** outlines this as a proposed decision pathway. We note that the evidence on whether completion lobectomy improves outcomes in patients found to have occult N1 disease is not settled (20, 21), and this recommendation is therefore framed as a default to be documented and reconsidered case by case rather than as an absolute requirement.

**Figure 3:**
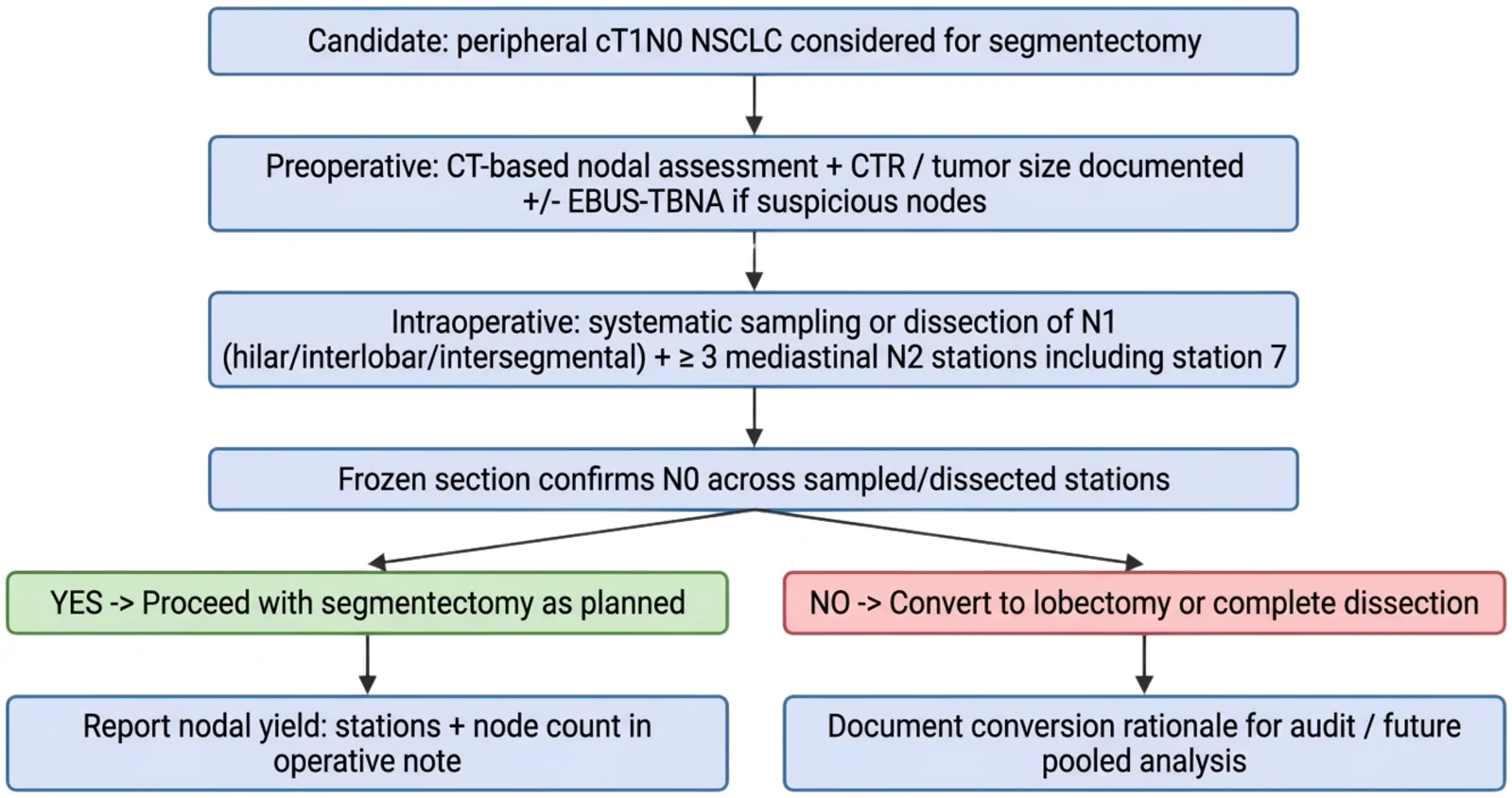
Proposed minimum nodal evaluation algorithm before offering segmentectomy outside a clinical trial setting. Each step is labeled with the source from which its requirement is drawn; the two reporting steps are recommendations of this review rather than external requirements. Created in BioRender. Magouliotis, D. (2026) https://BioRender.com/9v8slzx.

### 6.5 Nodal findings should be reported at the station level, not only as an aggregate count

The inconsistency with which adequacy has been defined and reported across the broader literature cannot be resolved retrospectively, but it can be prevented going forward. We recommend that segmentectomy series and operative notes alike report, at minimum, the specific stations sampled or dissected, the number of nodes recovered per station, whether N1 and N2 findings are distinguished, and which named standard the evaluation was benchmarked against.

### 6.6 Surgical approach should not substitute for meeting these criteria

Robotic assistance is associated with modestly higher nodal station yield than VATS (37, 38), but no evidence identified in this review demonstrates that approach choice alone reliably achieves adequate station coverage or improves risk-adjusted detection of occult disease. Approach should be selected on its own procedural merits, and nodal adequacy should be verified independently of it.

### 6.7 Adherence should be treated as a quality metric, not an assumption

Facility-level compliance with current nodal sampling guidelines correlates with institutional volume and academic status (17, 18), which means the rigor that validated segmentectomy in CALGB 140503 and JCOG0802 is not evenly distributed across the centers now offering it. Institutions adopting segmentectomy should audit their own station-level compliance prospectively, at the level of the individual surgeon and the individual case, and treat a shortfall as a modifiable process failure rather than as acceptable variation.

Two developments may change what adequate means over the next decade. The first concerns station 11. The interlobar nodes, including those lying along the interlobar arterial plane and at the bronchial sump, sit at the boundary of what a segmentectomy reliably reaches, and they were the commonest site of isolated lobar-segmental involvement in the anatomic series reviewed above (22). Making their dissection routine during both segmentectomy and lobectomy is a technically modest step that would reduce the chance of occult nodal disease going undetected, and it warrants prospective evaluation rather than continued reliance on retrospective mapping. The second concerns the learning curve. Most of the real-world data reviewed here describe operations performed during the 2010s, when segmentectomy was still being standardized and adopted unevenly across centers. As operative technique, intersegmental plane identification, and nodal protocols converge, recurrence and upstaging outcomes in the post-2020 era may not resemble those reported from the preceding decade. The adherence figures cited throughout this review should therefore be re-examined against contemporary cohorts before they are treated as a stable description of current practice.

**Figure 3** assembles these seven recommendations into a single proposed pathway. The population definition, the preoperative CTR threshold, the station-based intraoperative minimum, and the frozen-section conversion gate all trace directly to CALGB 140503 (10), JCOG0802 (11), CoC Operative Standard 5.8 (15), and the predictor studies discussed in Section 3 (23, 24). The two reporting steps at the foot of the pathway, documenting nodal yield at the station level and documenting the rationale for any conversion, are not drawn from an external source; they are recommendations 5 and 7 of this review, offered as a template rather than as a citation. No single published algorithm looks exactly like **Figure 3**. It is a synthesis of requirements that already exist separately across the trials and guidelines cited throughout this review, assembled into one pathway here.

## 7 Conclusion

CALGB 140503 and JCOG0802 did not establish that segmentectomy is unconditionally equivalent to lobectomy. They established that it is equivalent when nodal status is verified to a defined, protocol-enforced standard. Outside that context, segmentectomy and adequate nodal evaluation are too often treated as synonymous, when the literature and the real-world adherence data reviewed here suggest they should not be. Closing that gap does not require abandoning segmentectomy, nor does it require a new trial. It requires only that nodal evaluation performed outside a trial be held to the same explicit, checkable standard that made the results of the trials valid in the first place. Until reporting and practice converge on an explicit, station-based standard of the kind proposed here, the oncologic safety demonstrated in the randomized setting cannot be assumed to transfer automatically to every segmentectomy performed in its name. That standard is provisional. As standardized segmentectomy matures and contemporary series accumulate, both the adherence figures and the minimum proposed here should be re-audited rather than assumed to hold.
